# Improving Vancomycin Therapeutic Drug Monitoring With a Deep Learning–Based Two-Compartment Predictive Model: Development and Validation Study

**DOI:** 10.2196/81103

**Published:** 2026-06-01

**Authors:** Bingyu Mao, Ziqian Xie, Laila Rasmy, Masayuki Nigo, Degui Zhi

**Affiliations:** 1 McWilliams School of Biomedical Informatics, The University of Texas Health Science Center at Houston, 7000 Fannin, Suite 600, Houston, TX, United States, 1 713-500-3629, 1 713-500-3929; 2 Division of Infectious Diseases, Houston Methodist, Houston, TX, United States

**Keywords:** vancomycin, pharmacokinetics, compartmental models, recurrent neural network, electronic health records

## Abstract

**Background:**

Vancomycin is a widely used antibiotic that requires therapeutic drug monitoring (TDM) for optimized individual dosage. A deep learning–based model, pharmacokinetic recurrent neural network–1 compartment model (PKRNN-1CM), has shown the advantage of leveraging time-series electronic health record data for individualized estimation of vancomycin pharmacokinetic (PK) parameters. While 1-compartment PK models are commonly used because of their simplicity and previous trough-based clinical practices for dose adjustment, the pre–deep learning literature suggests the superiority of 2-compartment models.

**Objective:**

This study introduces the pharmacokinetic recurrent neural network–2 compartment model (PKRNN-2CM), a novel deep learning–based model designed to improve vancomycin TDM by integrating a 2-compartment PK framework.

**Methods:**

PKRNN-2CM combines recurrent neural network–driven PK parameter estimation with a 2-compartment PK model to predict vancomycin concentration trajectories. Training on both simulated data and real-world electronic health record data allows for a comprehensive evaluation of its performance.

**Results:**

Experiments based on simulated data highlight PKRNN-2CM’s superiority over the simpler 1-compartment model, PKRNN-1CM, in predicting vancomycin concentration measurements (root mean square error 3.04 vs 4.50). Application to a real dataset from 5483 patients showcases significant improvement over PKRNN-1CM (root mean square error 5.55 vs 5.65; 2-sample 2-tailed unpaired *t* test; *P*=.01), with potential further gains expected with nontrough level measurements. Our simulation also indicates that PKRNN-2CM offers a better estimate of the average area under the concentration-time curve to minimum inhibitory concentration ratio, a more clinically relevant measure.

**Conclusions:**

PKRNN-2CM is an important improvement in vancomycin TDM, demonstrating enhanced accuracy and performance compared to the PKRNN-1CM model. This deep learning model holds potential for future individualized vancomycin TDM optimization and broader applications in diverse clinical scenarios.

## Introduction

Therapeutic drug monitoring (TDM) is necessary for optimizing individual dosage regimens, particularly for drugs with narrow therapeutic ranges and high pharmacokinetic (PK) variability, such as vancomycin [1,2]. Traditional vancomycin TDM methods include trough monitoring, linear regression, population PK (popPK), and Bayesian estimation [1]. The most recent national guidelines recommend individualized dosing guided by Bayesian methods for TDM, but they may not effectively cover diverse patient populations and are often unsuitable for patients with unstable clinical conditions [3,4]. Furthermore, these models incorporate a limited number of patient-specific variables, while neglecting other factors that could enhance predictions [3]. Deep learning models have shown significant potential in PK-related modeling [5,6], especially for concentration prediction, as they can handle large datasets and complex information. Neural ordinary differential equations (ODEs) [7] have been applied to PK prediction, showing better performance in predicting untested treatment regimens compared to traditional nonlinear mixed-effects models. Building on this, the deep compartment model used neural networks to predict parameters for an ODE, further improving drug concentration predictions and enabling effective TDM, even with small training datasets [8]. Additionally, recurrent neural networks (RNNs) prove to be well-suited for modeling time-series electronic health record (EHR) data [9] due to their capacity to analyze sequences of time-related events. When it comes to TDM, which inherently involves irregularly sampled and noisy time-series EHR data, RNNs become a fitting choice. As an example, the pharmacokinetic recurrent neural network (PKRNN) model [10] is an autoregressive RNN model with a PK prediction head, which outperforms a Bayesian vancomycin TDM model. However, the PKRNN model only used a 1-compartment PK model. It is unclear if multicompartment PK models will further improve PKRNN.

Here, we extend their work, aiming to improve the performance of the pharmacokinetic recurrent neural network–1 compartment model (PKRNN-1CM) by incorporating a 2-compartment PK prediction head. The determination of the number of compartments in developing popPK models is important for describing the PK of drugs [11]. While 1-compartment models (1CMs) assume vancomycin distributes evenly throughout the body right after the infusion, multicompartment models exhibit rapid initial distribution followed by slower elimination, offering a more realistic representation of vancomycin distribution. Previous work reports that vancomycin PK has been described using 1CMs, 2-compartment models (2CMs), and 3-compartment models, while most Bayesian approaches use 1CMs or 2CMs [11]. Their preference for 2CMs is further supported by studies indicating their superiority in achieving higher accuracy and lower bias when predicting vancomycin concentrations [12]. 2CMs can provide more accurate predictions, especially for critically ill patients with non–steady-state kidney function [13,14]. Additional evidence from studies [11,15,16] shows the advantages of 2CMs over 1CMs. Detailed additional background information can be found in Multimedia Appendix 1.

Despite convincing evidence supporting the superiority of 2-compartment PK models, the 1CM is still widely adopted in clinical practice. This preference may be attributed to its simplicity and the reliance on previous trough-based common practices for dose adjustment. We hypothesize that increasing the number of nontrough level measurements of the concentration-time curve in the development of a 2-compartment PK model will enhance its performance, resulting in significantly improved prediction accuracy compared to a 1-compartment PK model. This hypothesis is grounded in the observations of [11], who noted that, for a given patient, the 2 curves differ the most at the peak level and become smaller the closer they are to the trough level. Testing this hypothesis requires a dataset rich in nontrough level measurements, which is challenging given the sparse real-world datasets. Therefore, we turn to simulation as a valuable tool, providing a controlled environment to explore different sampling strategies and thoroughly examine the impact of various factors, such as the density and timing of measurements, on prediction accuracy to comprehensively assess and refine the developed 2-compartment PK model. This study innovatively uses actual patient information in the simulation, ensuring comparability with real-world datasets and distinguishing it from existing works that use simulated data for model comparison.

This study aims to develop an RNN-based 2-compartment predictive model (pharmacokinetic recurrent neural network–2 compartment model [PKRNN-2CM]) for vancomycin TDM. It is anticipated that the combination of an RNN and a 2-compartment PK model will provide better predictions of vancomycin concentrations than the PKRNN-1CM model, especially when there is a sufficient number of observations occurring during nontrough levels. The proposed model is evaluated using both real-world clinical data and a simulation framework based on actual EHR information to assess performance under diverse sampling and clinical conditions.

## Methods

### Data Description

This study used the same dataset as [10], consisting of 5483 patients with 8689 encounters in total, which was obtained from an EHR data warehouse containing information regarding encounter-level administrative data collected from Memorial Hermann Health System (MHHS), a large health care system based in Houston, Texas, United States. Patients aged more than 18 years who received at least 1 dose of vancomycin during the period from August 2019 to March 2020 were included in the study. This study excluded patients who underwent renal replacement therapy and patients with inappropriate timing of vancomycin levels. Deidentification of the extracted cohort was conducted to protect the privacy and security of the patient data. Similar to a study by Nigo et al [10], the model was trained and evaluated only using encounters with at least 1 serum vancomycin level measured after the first vancomycin dose.

The PKRNN-2CM model is designed to use variables that are routinely collected in EHR. The EHR data for this study were extracted at the encounter level, including 30 laboratory tests, 5 vital signs, 324 types of medications, and demographic information. For medication features, we represented drugs using their simple generic names rather than pharmacologic classes. This choice was based on our prior work [17], which showed that finer clinical granularity can improve model performance in high-dimensional EHR models. Using generic names preserves clinically meaningful distinctions without introducing the excessive detail associated with brand names or dose-specific entries. In addition, medication variables were encoded using embedding representations, which help mitigate data sparsity while retaining relevant clinical information. The variables included in this study are commonly measured in clinical practice, particularly complete blood counts and basic metabolic panels.

Out of the 40 continuous variables, some laboratory tests, such as serum calcium level, phosphorus, total bilirubin, total protein, serum magnesium, and nucleated red blood cells, may occasionally have missing values. However, these laboratory tests are typically measured at least once during hospitalization when bacterial infections are suspected and vancomycin is used. Other variables included in the study are demographic data and vital signs, which are also routinely collected in hospitals. The 8 categorical variables are used to create an embedding vector for medications. In the same manner as described by Nigo et al [10], for each encounter, the start time was determined by the earliest record time, while the end time was determined by the time stamp of the last vancomycin concentration. Vancomycin and other medication administration, vancomycin and other laboratory measurements, and the end of the day were used as events to update the state of the PKRNN-2CM model. Within the MHHS dataset, the infusion duration of vancomycin is dosage-dependent: 1000 mg is infused over 1 hour, 1001 to 1500 mg over 1.5 hours, 1501 to 2000 mg over 2 hours, and doses exceeding 2000 mg over 2.5 hours. For simplicity, a uniform infusion rate of 1 g/h was adopted. This standard rate is used in MHHS as a default rate to prevent acute reactions such as red-man syndrome. While individual infusion rates were not available in the MHHS dataset, the PKRNN-2CM model can be adjusted to incorporate various infusion rates, making it adaptable to different clinical settings. We used forward filling and mean imputation to handle missing covariates. Continuous variables were standardized using *z* scores.

### Model Architecture and PK Modeling

**Figure 1.**
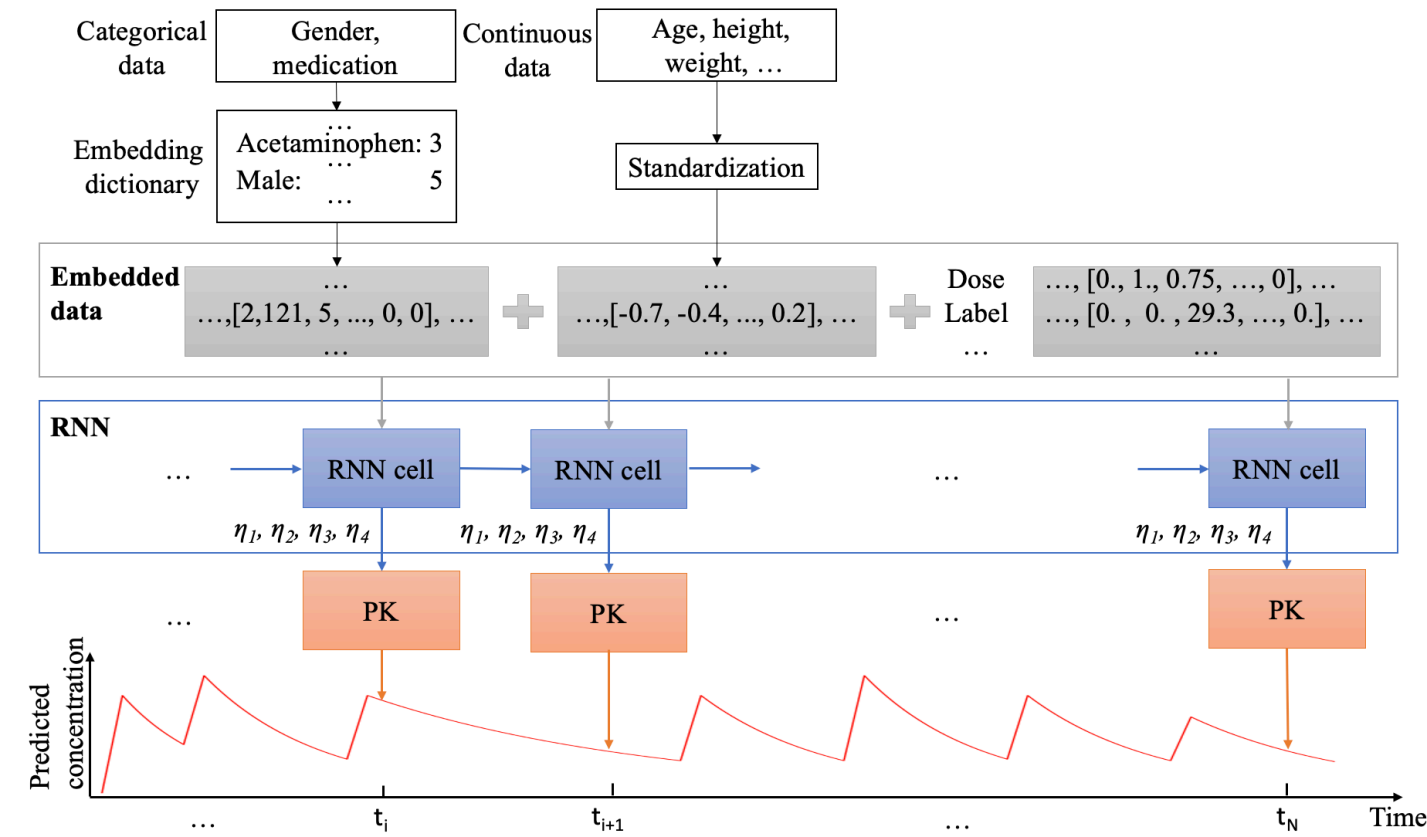
Pharmacokinetic recurrent neural network–2 compartment model (PKRNN-2CM) architecture. PK: pharmacokinetic; RNN: recurrent neural network.

The PKRNN-2CM model, similar to the PKRNN-1CM model [10], is an autoregressive RNN built on EHR data, requiring minimal manual input, and providing real-time concentration predictions by automatically adjusting to reflect subtle changes. The PKRNN-1CM model consists of 3 components that enable the vancomycin predictions to be evaluated at any time point. First, a code embedding layer is used to convert information from the EHR into the PKRNN-1CM model for every time step. Next, an RNN layer is applied to predict vancomycin elimination rates and compartment volumes, and finally, based on the output from the RNN layer, a 1-compartment PK layer uses the PK equation, which is an ODE, to compute the predicted vancomycin concentration. For the PKRNN-2CM model, we extend the 1-compartment PK model to a 2-compartment PK model. A schematic representation of the structure of the PKRNN-2CM model can be found in Figure 1. The input to the model is the time-series EHR data after data preprocessing, which contains 8-dimensional embedding derived from categorical data that includes all 911 different medication codes of 324 medications (Multimedia Appendix 1 shows the generic names of medications used in the model) and 40D continuous data that includes time, time difference, vancomycin dose, vancomycin serum concentration measurements, laboratory tests, and demographic information (Multimedia Appendix 1 shows all the continuous variables included in the model). For every time step, the RNN layer predicts 4 parameters *η*_1_, *η*_2_, *η*_3_, and *η*_4_ [18] that are related to the PK parameters. After that, the PK layer based on the 2-compartment PK model uses an ODE to calculate the estimated PK parameters *CL* (clearance for the central compartment), *Q* (intercompartmental clearance), *V*_1_ (volume of the central compartment), and *V*_2_ (volume of the peripheral compartment), then uses the estimated PK parameters to predict the vancomycin concentration.

**Figure 2.**
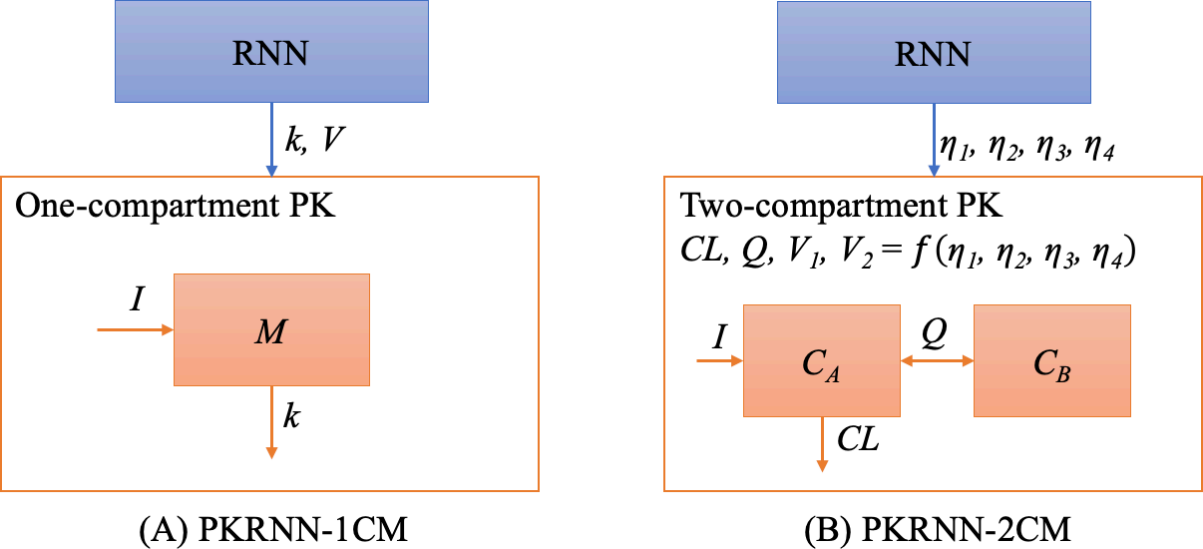
The main difference between pharmacokinetic recurrent neural network–1 compartment model (PKRNN-1CM) and pharmacokinetic recurrent neural network–2 compartment model (PKRNN-2CM). (A) The recurrent neural network (RNN) and pharmacokinetic (PK) layers of PKRNN-1CM, where *k* is the elimination rate, *V* is the volume distribution, *I* is the infusion rate, and *M* is mass of the compartment. (B) The RNN and PK layers of PKRNN-2CM, where *η*_1_, *η*_2_, *η*_3_, and *η*_4_ are PK parameters that satisfy a multivariate Gaussian distribution; *CL* is the clearance of the central compartment; *Q* is the intercompartmental clearance; *C_A_* is the concentration of the central compartment; and C_*B*_ is the concentration of the peripheral compartment.

PKRNN-2CM differs from PKRNN-1CM in two aspects: (1) it uses additional PK parameters and different initial values; and (2) it uses a first-order ODE system with 2 ODEs to calculate vancomycin concentrations. As Figure 2 shows, there are 2 PK parameters in the PKRNN-1CM model, elimination rate *k* and volume of distribution *V*, while the PKRNN-2CM model has 2 clearances, central (*CL*) and intercompartmental (*Q*), and 2 volumes, *V*_1_ and *V*_2_ representing the central and peripheral compartments, respectively. Furthermore, empirically we found that the performance improved by removing the constraints of mass conservation as in PKRNN-1CM. Instead, in PKRNN-2CM we make the concentration continuous, which we believe is better because the model is allowed to correct its own prediction error in a smoother way.

Based on a study by Lim et al [18], the 4 PK parameters *CL*, *Q*, *V*_1_, and *V*_2_ can be described by 4 related parameters *η*_1_, *η*_2_, *η*_3_, and *η*_4_ that follow a multivariate normal distribution (MVN). The RNN layer in the PKRNN-2CM predicts *η*_1_, *η*_2_, *η*_3_, and *η*_4_ where the initial values are determined according to Lim et al’s [18] study:


(1) $$\left[\eta_{1},\;\eta_{2},\;\eta_{3},\eta_{4}\right]∼MVN\left(\left[0,\;0,\;0,\;0\right],\;\mathrm{diag}\left(\left[0.120,\;0.149,\;0.416\right]\right)\right)$$

as opposed to PKRNN-1CM which uses RNN to predict *k* and *V* directly. In the PK layer, *η*_1_, *η*_2_, *η*_3_, and *η*_4_ are used to calculate PK parameters following the equations below, where the content in brackets indicates the unit:


(2) $$V_{1}=33.1{∙e}^{\eta 1}(L)$$

(3) $$V_{2}=48.3{∙e}^{\eta 4}\left(L\right)$$

(4) $$Q=6.99\cdot e^{\eta 3}\;\left(L/hr\right)$$

(5) $$CL=0.0396\cdot CrCL\cdot e^{\eta 2}\;\left(L/hr\right)$$

where *CrCL* is the creatinine clearance calculated from the Cockcroft-Gault equation:


(6) $$CrCL=\frac{\left(140-age\;(yr))\times weight\;(kg\right)}{72\times serum\;Cr\;(mg/dL)}\left(\times 0.85\;for\;women\right)$$

The vancomycin concentration can then be calculated using the ODE system below:


(7) $$\frac{dC_{A}}{dt}=\frac{I}{V_{1}}-\frac{CL}{V_{1}}C_{A}-\frac{Q(C_{A}-C_{B})}{V_{1}}\;\frac{dC_{B}}{dt}=\frac{Q(C_{A}-C_{B})}{V_{2}}$$

Where *C_A_* and C_*B*_ are the concentrations in central and peripheral compartment (mg/L).

A closed-form solution to this ODE can be found in Multimedia Appendix 2.

### Simulation Framework and Design

The simulation was designed to be a valuable tool to guide the development and deployment of our PKRNN-2CM model by bridging the gap between the constraints of real-world data and the requirements of a comprehensive and reliable predictive system. Through this approach, we can address the limitations of sparse real-world datasets and gain deeper insights into the behavior of the PKRNN-2CM model in comparison to the PKRNN-1CM model. To ensure that the simulated dataset was comparable to the original real-world dataset, the simulation was based on actual patient information such as medications and laboratory results. As much patient information as possible was used in the simulation to guarantee the similarity between the simulated datasets and the original real-world dataset, with the only simulated data being the vancomycin measurements. We first trained a PKRNN-2CM model using real-world data, resulting in the PKRNN-2CM–derived model, which was then used to simulate vancomycin concentrations. This PKRNN-2CM–derived model was set as the “ground truth” to generate measurements for the simulated datasets. We then applied either the PKRNN-1CM or PKRNN-2CM model to the simulated data to learn the parameters, resulting in the PKRNN-1CM–estimated or PKRNN-2CM–estimated models. Although the PKRNN-2CM–derived and PKRNN-2CM–estimated models share the same architecture, they are trained independently without exchanging information. In summary, the PKRNN-2CM–derived and PKRNN-2CM–estimated models operate independently, with the only connection being that the PKRNN-2CM–estimated model uses the predicted concentrations from the PKRNN-2CM–derived model as simulated measurements.

The simulation used in this study involves the use of vancomycin concentrations predicted by the PKRNN-2CM–derived model as labels for testing the performance of the PKRNN-1CM–estimated and PKRNN-2CM–estimated models based on different types of simulation.

**Figure 3.**
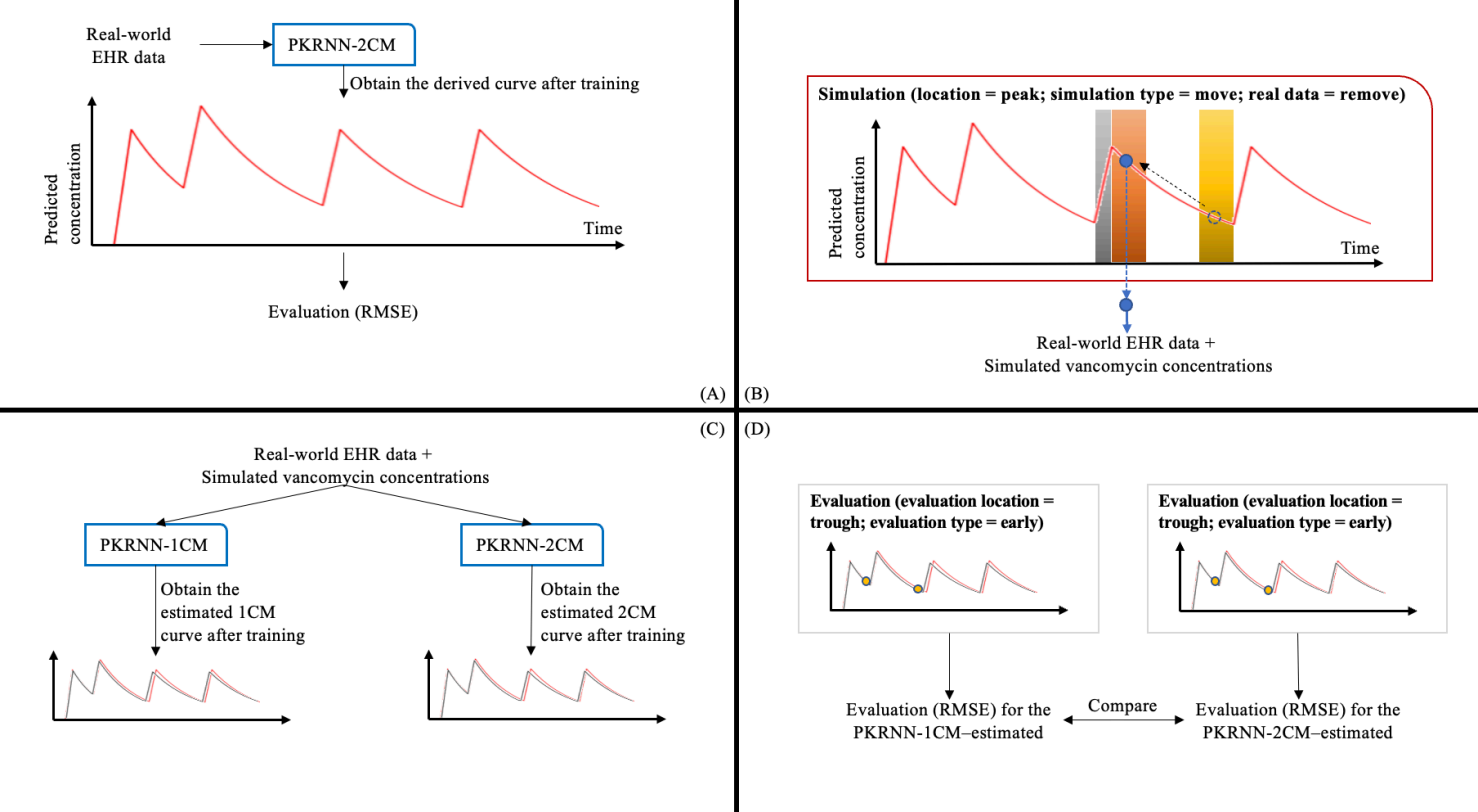
Simulation process. (A) Real-world EHR data are used to train the PKRNN-2CM–derived model and evaluate prediction performance using RMSE. (B) The trained PKRNN-2CM–derived model generates simulated vancomycin concentration data under selected simulation settings. (C) PKRNN-1CM–estimated and PKRNN-2CM–estimated models are trained using the simulated dataset. (D) The estimated models are evaluated and compared using root mean square error (RMSE) under different evaluation settings. 1CM: 1-compartment model; 2CM: 2-compartment model; EHR: electronic health record; PKRNN-1CM: pharmacokinetic recurrent neural network–1 compartment model; PKRNN-2CM: pharmacokinetic recurrent neural network–2 compartment model.

Figure 3 is a schematic diagram showing the detailed simulation and evaluation process. In step 1, the real-world time-series EHR data are loaded into the system. In step 2, the PKRNN-2CM–derived model is trained on real data. In step 3, simulation is set to false and model performance is assessed through the calculation of root mean square error (RMSE), comparing observed and predicted concentrations. In step 4, simulation is set to true, and the real data are removed, and predicted concentrations from the PKRNN-2CM–derived model are used to generate a simulated dataset based on the simulation options. In step 5, the PKRNN-1CM–estimated or PKRNN-2CM–estimated model is trained on the simulated data. Step 6 evaluates the estimated model’s performance using RMSE. Finally, a comparative analysis of RMSEs between PKRNN-1CM–estimated and PKRNN-2CM–estimated models measures their efficacy in different scenarios. A detailed simulation process overview can be found in Multimedia Appendix 1.

### Implementation Details

To establish the framework for our model implementation, we meticulously configured the code embedding and the RNN layers, and also chose training hyperparameters and optimization techniques for optimal performance. In configuring the code embedding layer, categorical data are embedded into 8D vectors. For the initialization of the embedding layer, weights were established using a Gaussian distribution. Each time step involves the input of a 48D vector (40+8) into the RNN layer, comprising embedded categorical data (8) and normalized continuous data (40). The RNN layer used a single-layer gated recurrent unit (GRU) with a hidden size of 64. The output layer is characterized by a linear layer of size (64, 4), mapping the GRU’s hidden layer to the parameters *η*_1_, *η*_2_, *η*_3_, and *η*_4_ at each time step. For model training, the Adamax optimizer is used with a learning rate and weight decay set at 1 × 10⁻² and 0.2, respectively. The training minibatch size is configured at 50, and an early stopping mechanism, with “patience” set to 10, is implemented to mitigate the risk of overfitting. The mean squared error serves as the loss function for training the model. Additionally, 2 regularization mechanisms were incorporated. First, a penalty was imposed on the deviation of the predicted parameters *η*_1_, *η*_2_, *η*_3_, and *η*_4_ from the prior multivariate Gaussian distribution. The second regularization term uses the L2 norm of the first-order difference to discourage abrupt changes in the output of the RNN layer.

For the simulation implementation, several datasets were generated from the PKRNN-2CM–derived model based on different simulation options. In the “peak” datasets, a measurement was simulated after the vancomycin administration. The time intervals between administration and measurement were dose-dependent; the simulated measurements were made after 2 hours if the dose was less than or equal to 1000 mg, or 3 hours if the dose was greater than 1000 mg. The “trough” datasets simulated each measurement 1 hour before the vancomycin administration.

For consistency and comparability, all models were evaluated using the same dataset and criteria. Depending on patient identification, the data were divided into training, test, and validation sets in a ratio of 50:30:20. The performance of PKRNN-2CM and PKRNN-1CM was compared on both the original MHHS dataset and the simulated datasets using RMSE. A 2-sample 2-tailed *t* test was used to compare the model performance between PKRNN-1CM and PKRNN-2CM. Additionally, we ran the PKRNN-1CM–estimated or PKRNN-2CM–estimated model with different simulation options to evaluate how the estimated models captured the area under the concentration curve. This study was conducted with Python 3.8 (Python Software Foundation) using the *PyTorch* 1.9.0 library. All experiments were run on an Nvidia A100 80GB GPU. The training process takes less than 60 seconds per epoch, and evaluation and testing take less than 30 seconds per epoch.

A detailed code repository for reproducing the experiment can be found on GitHub [19].

### Ethical Considerations

This study was approved by the institutional review board at The University of Texas Health Science Center at Houston (approval: HSC-MS-19‐1011) and was considered exempt from consent due to the retrospective nature of the study and use of deidentified electronic health record data. All data were handled in compliance with institutional policies and applicable regulations for the protection of patient privacy and confidentiality. No identifiable patient information was included in the analysis or reported in this study

## Results

### Data Characteristics

Table 1 summarizes the baseline characteristics of the study cohort. A total of 5483 patients were included in the analysis.

**Table 1.** Descriptive statistics for the study cohort (N=5483).

Characteristics	Values
Basic characteristics	
Encounters, n (%)	8689 (100)
Weight (kg), median (IQR)	82.9 (65.5‐101.6)
Height (cm), median (IQR)	172 (165.1‐181.1)
Demographics	
Age (y), median (IQR)	61 (48‐73)
Sex, n (%)	
Male	3069 (55)
Race and ethnicity, n (%)	
African American	1069 (19.5)
Asian	83 (2)
Hispanic	783 (14.2)
Non-Hispanic	3905 (71.2)
White	2003 (36)

### Model Evaluation on Real-World Data

Table 2 shows that the PKRNN-2CM model exhibited better performance compared to the PKRNN-1CM model (a 2-sample 2-tailed *t* test; *P*=.01) on the MHHS datasets. A Bayesian vancomycin therapeutic drug monitoring (VTDM) model [18] was implemented as the baseline here, with PK parameters updated after each measurement using maximum a posteriori estimation. The VTDM model performed worse than any of the PKRNN models.

**Table 2.** Results of the pharmacokinetic recurrent neural network–1 compartment model (PKRNN-1CM) and the pharmacokinetic recurrent neural network–2 compartment model (PKRNN-2CM) on the Memorial Hermann Health System (MHHS) dataset.

Model	Average RMSE^a^ (SD)^b^	*P* value
PKRNN-1CM	5.65 (0.06)	—^c^
PKRNN-2CM	5.55 (0.04)	.01
VTDM^d^	6.38^e^	—

a RMSE: root mean square error.

b SD over 5 repeats.

c Not applicable.

d VTDM: vancomycin therapeutic drug monitoring.

e Note that the SD for VTDM is not applicable because it is deterministic and evaluated once.

### Simulation Results

Simulation results were reported using average RMSEs from a common test set. For each dataset simulated from the PKRNN-2CM–derived model, we tested and compared the model performance of the PKRNN-1CM–estimated and PKRNN-2CM–estimated models. Table 3 presents the summarized simulation results.

**Table 3.** Results of the pharmacokinetic recurrent neural network–1 compartment model (PKRNN-1CM) and the pharmacokinetic recurrent neural network–2 compartment model (PKRNN-2CM) on the simulated dataset.

Measurement	Average RMSE^a^ (SD)^b^		*P *value
	PKRNN-1CM–estimated	PKRNN-2CM–estimated	
Measurements for half of the doses			
Simulation location			
Peak^c^	7.06 (0.71)	4.27 (0.41)	<.001
Trough^d^	9.71 (1.43)	3.61 (0.14)	<.001
Both^e^	5.65 (0.44)	2.50 (0.25)	<.001
Measurements for all doses			
Simulation location			
Peak^c^	4.50 (0.13)	3.04 (0.21)	<.001
Trough^d^	8.46 (2.20)	2.72 (0.19)	<.001
Both^e^	3.85 (0.33)	1.94 (0.06)	<.001

a RMSE: root mean square error.

b SD over 5 repeats.

c Peak: 2 or 3 hours post–every infusion, 2 hours if infusions are ≤1000 mg, 3 hours if infusions are >1000 mg.

d Trough: 1 hour pre–every infusion.

e Both: 2 measurements (peak and trough) for every infusion.

Table 3 shows the performance of the PKRNN-1CM and PKRNN-2CM on the simulated dataset. Overall, models trained on simulated measurements achieved lower RMSE than those trained on real-world data, and performance improved with increased measurement frequency. Given a fixed measurement budget, combining peak and trough observations consistently outperformed sampling only peak or trough levels, highlighting the benefit of balanced sampling strategies. In most settings, PKRNN-2CM showed slightly lower variability than PKRNN-1CM, indicating more stable performance. The VTDM model performed poorly on the simulation data, likely due to its inflexibility in adapting to the dynamic PK parameters generated by PKRNN-2CM–derived data. The results of VTDM on the simulated data are shown in Multimedia Appendix 1.

**Figure 4.**
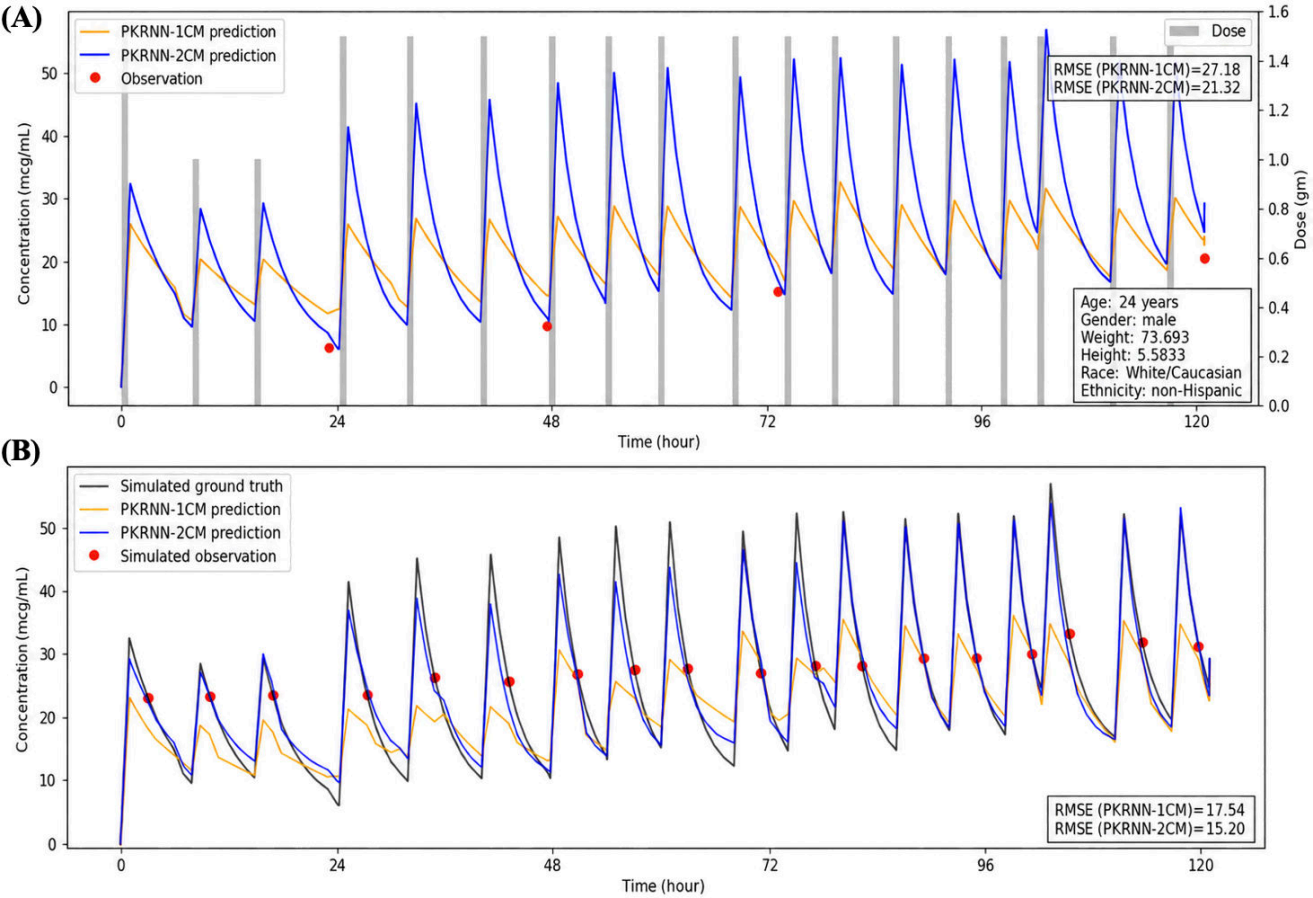
Model performance for scenario 1. (A) Real-world patient data. (B) Simulated patient data. The PKRNN-2CM model achieved lower RMSE values in both settings. PKRNN-1CM: pharmacokinetic recurrent neural network–1 compartment model; PKRNN-2CM: pharmacokinetic recurrent neural network–2 compartment model; RMSE: root mean square error.

**Figure 5.**
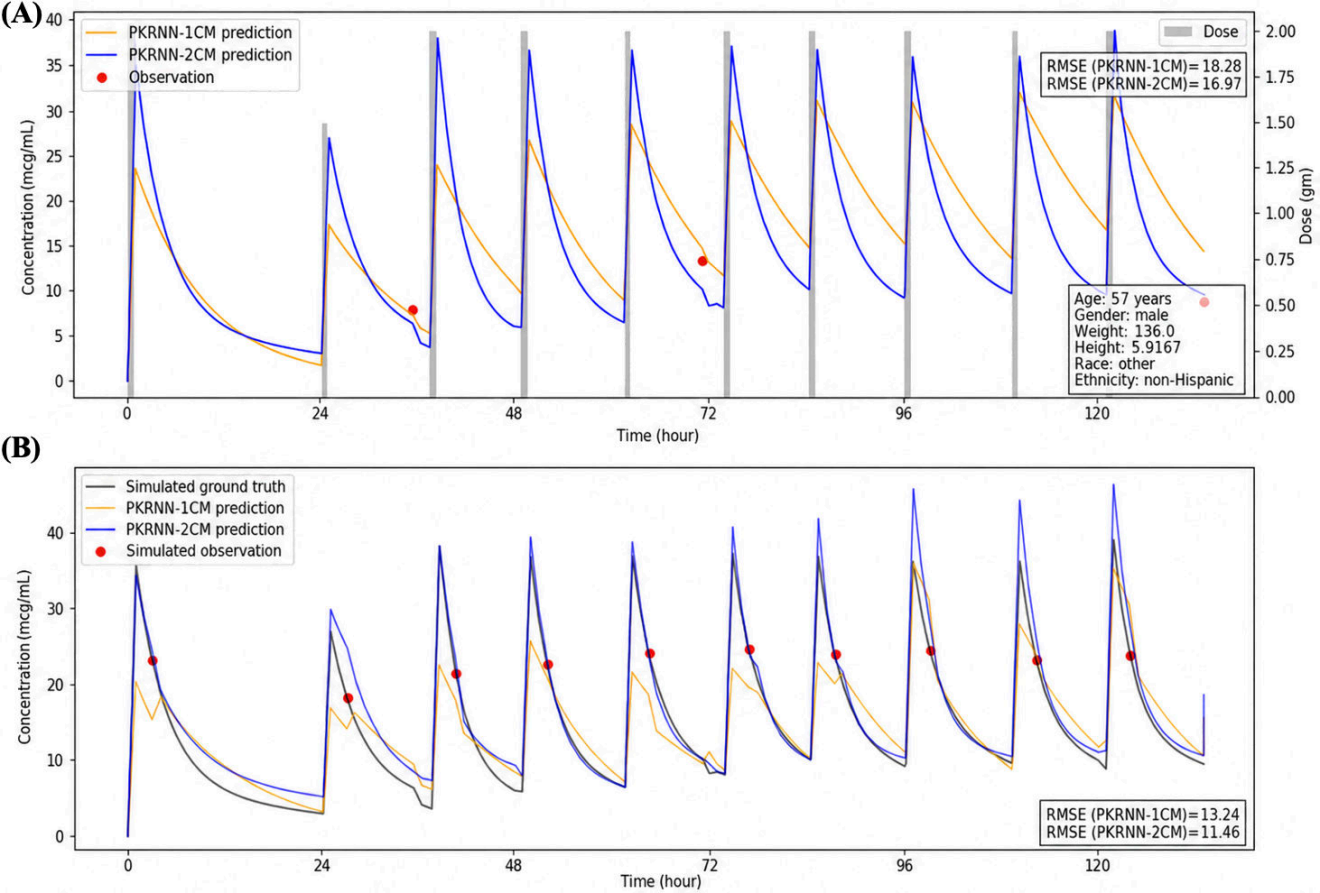
Model performance for scenario 2. (A) Real-world patient data. (B) Simulated patient data. Both models showed similar performance on real-world data, while the PKRNN-2CM model achieved lower RMSE on simulated data. PKRNN-1CM: pharmacokinetic recurrent neural network–1 compartment model; PKRNN-2CM: pharmacokinetic recurrent neural network–2 compartment model; RMSE: root mean square error.

**Figure 6.**
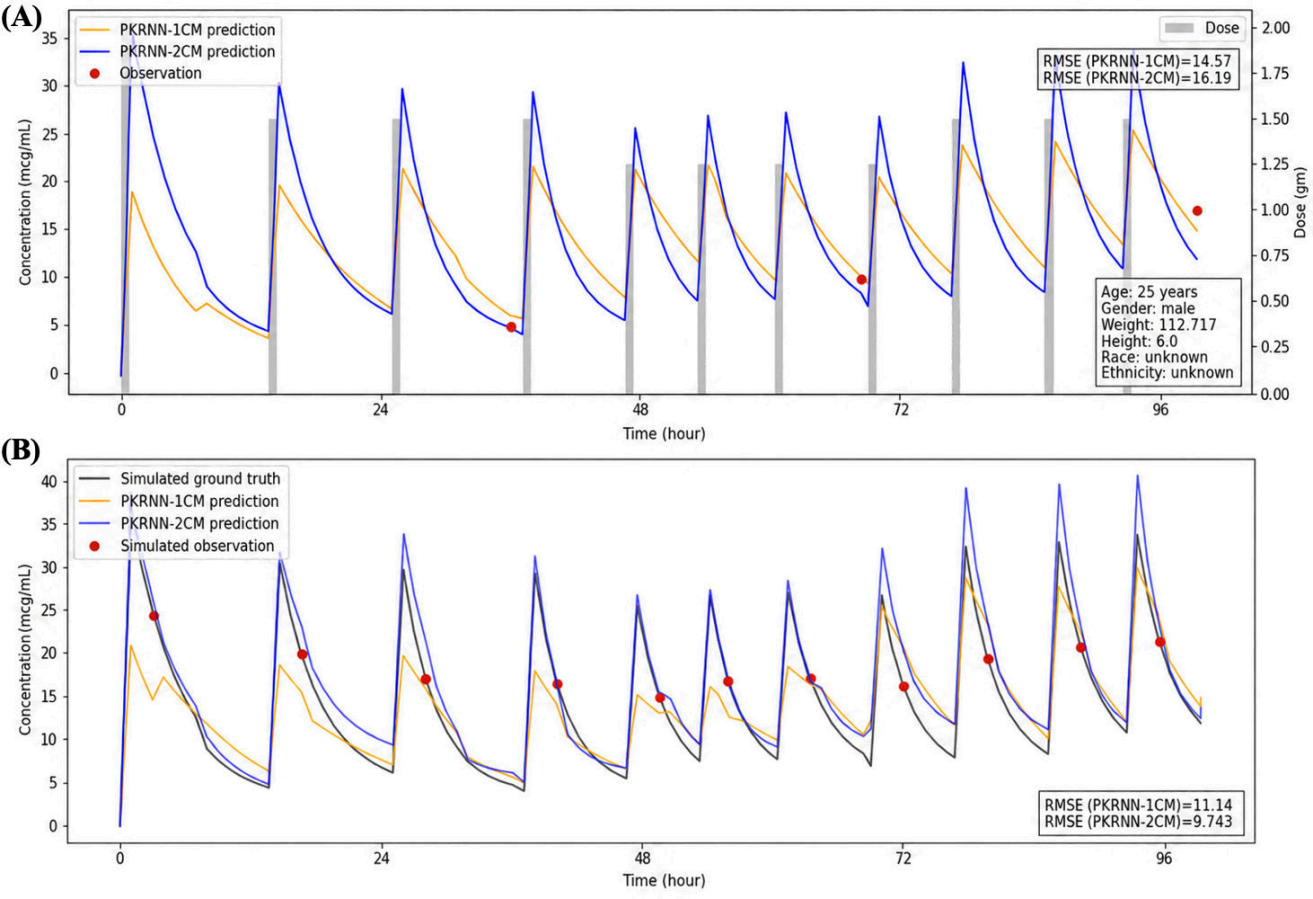
Model performance for scenario 3. (A) Real-world patient data. (B) Simulated patient data. The PKRNN-1CM model performed slightly better on real-world data, whereas the PKRNN-2CM model achieved lower RMSE on simulated data. PKRNN-1CM: pharmacokinetic recurrent neural network–1 compartment model; PKRNN-2CM: pharmacokinetic recurrent neural network–2 compartment model; RMSE: root mean square error.

Figures4^6^ present 3 randomly selected patient scenarios to show model behavior across different clinical settings. Each figure compares the PKRNN-1CM and PKRNN-2CM models using both real data (top panels) and simulated data (bottom panels). In Figure 4, the PKRNN-2CM model shows a clear advantage, achieving lower RMSEs in both real and simulated settings (real: 27.18 vs 21.32; simulated: 17.54 vs 15.20). In Figure 5, the 2 models perform similarly on real data, while the PKRNN-2CM–estimated model provides better accuracy on the simulated dataset (13.24 vs 11.46). In Figure 6, the PKRNN-1CM model slightly outperforms the PKRNN-2CM model on the real data (14.57 vs 16.19), whereas the PKRNN-2CM–estimated model performs better in the simulated scenario (11.14 vs 9.74).

Across Figures 4-6, predicted area under the concentration-time curves (AUCs) from the PKRNN-1CM model exhibited more abrupt transitions, particularly during the postinfusion exponential decay phase. These arise because the 1CM corrected its trajectory abruptly when new observations deviated from prior predictions. In contrast, the PKRNN-2CM model produced smoother curves by updating its state more gradually as new information became available. The curve behavior reflected the prospective plotting strategy, where earlier predictions were not retroactively adjusted. This emphasized real-time diagnostic interpretability over retrospective smoothing.

Table 4 compares the time-averaged, dose-normalized AUC/minimum inhibitory concentrations (MICs) calculated from different simulation options. The ground truth was derived from the PKRNN-2CM–derived model. The results indicate that the PKRNN-2CM–estimated model can provide a closer estimate of AUC/MIC compared with the PKRNN-1CM–estimated model. Additionally, for both models, predictions from combined peak and trough measurements yield smaller differences from the ground truth compared to peak or trough measurements. Thus, mixing peak and trough measurements can improve the accuracy of the predicted concentration-time curves for both estimated models. As before, given a fixed measurement budget, obtaining both peak and trough levels is superior to sampling measurements only at the peak or trough level across the doses.

**Table 4.** Time-averaged dose-normalized area under the concentration-time curve (AUC)/minimum inhibitory concentration (MIC) comparison for simulated datasets.

Measurements	RMSE^a^ of time-averaged dose-normalized AUC or MIC^b^ (SD)^c^		*P *value
	PKRNN-1CM^d^–estimated	PKRNN-2CM^e^–estimated	
Measurements for half doses			
Simulation location			
Peak^f^	55.97 (2.15)	36.65 (2.07)	<.001
Trough^g^	72.62 (7.70)	42.00 (2.67)	<.001
Both^h^	55.55 (2.60)	25.74 (1.04)	<.001
Measurements for all doses			
Simulation location			
Peak^f^	52.15 (4.69)	27.80 (0.73)	<.001
Trough^g^	63.79 (12.10)	30.57 (1.46)	<.001
Both^h^	45.49 (3.47)	21.70 (1.03)	<.001

a RMSE: root mean square error.

b Time-averaged, dose-normalized AUC/MIC: area under the concentration-time curve to minimum inhibitory concentration ratio, averaged over the duration and normalized by the maximum dose.

c SD over 5 repeats.

d PKRNN-1CM: pharmacokinetic recurrent neural network–1 compartment model.

e PKRNN-2CM: pharmacokinetic recurrent neural network–2 compartment model.

f Peak: 2 or 3 hours post–every infusion, 2 hours for infusions ≤1000 mg, 3 hours for infusions >1000 mg.

g Trough: 1 hour pre–every infusion.

h Both: 2 measurements (peak and trough) for every infusion.

## Discussion

The results demonstrate a significant improvement in the PKRNN-2CM model’s performance compared with the PKRNN-1CM model in real-world data. The results from the simulation studies documented not only the overall superiority of the PKRNN-2CM model over the PKRNN-1CM model but also highlighted a greater performance gap in scenarios involving nontrough level measurements. PKRNN-2CM is a deep-learning model that combines an RNN for vancomycin PK parameter estimation with a 2-compartment PK model for generating concentration trajectories, offering an innovative approach for individualized vancomycin TDM using time series EHR data.

This study demonstrated a superior predictive accuracy of a 2-compartment PK model over the 1-compartment PK model using a large real-world dataset. Current experiments that compare the 1-compartment and 2-compartment PK models for prediction tasks have either used small datasets with dense sampling [11] or relied on simulated data that did not incorporate real-world patient information, such as using a single standard simulated patient or generating simulated populations without any real-world data [20,21]. In contrast, the approach bridges this gap in 2 ways. First, we simulated densely sampled data based on real-world patient characteristics from a large clinical dataset, showing the superiority of the PKRNN-2CM model. Second, we evaluated both models on the original real-world dataset with sparse measurements and again found that the PKRNN-2CM model delivered superior performance compared with PKRNN-1CM. These findings highlight the robustness and practical use of the PKRNN-2CM model, especially in realistic clinical settings where data are often sparse and heterogeneous.

One of the main contributions of this study is our simulation framework, which offers a new approach by comparing the performance of 1-compartment and 2-compartment PK models based on real-world data. Many existing studies using simulated data to compare the 1-compartment and 2-compartment PK models either use 1 simulated standard patient to evaluate different model performances [20] or sample all the patient information for a population distribution [21]. The simulation in this work strives for a more realistic representation by using as much real patient information as possible, with the only simulated components being the measurements. By grounding our simulation in real-world patient demographics, medications, and laboratory results, we ensure that the simulated datasets retain a level of reality to the complexities observed in realistic clinical scenarios.

The simulation framework, with its diverse simulation and evaluation options, enables the evaluation of PK models in real-world scenarios. This simulation framework can be used to investigate and assess different sampling or measurement strategies in distinct clinical settings and applications. In addition to different simulation options, our evaluation strategy for the simulation has the advantage of overcoming the limitations imposed by the availability of real-world measurements. In real-world scenarios, the evaluation of PK model performance is constrained by the availability of real measurements, leaving gaps in understanding the model’s efficacy at specific levels of the concentration-time curve. However, the inclusion of different evaluation options in our simulation framework partially circumvents these constraints, enabling an evaluation of PK model performance across various scenarios. Building on this framework, the model design centered on the RNN component because of its ability to model irregular, noisy time-series EHR data. We note, however, that a direct comparison with alternative architectures (eg, multilayer perceptron or convolutional neural network) was not performed in this study. The design choice was guided by previous work demonstrating the suitability of RNNs for sequential clinical data.

There are some similarities between the PKRNN-2CM model and previously published popPK and Bayesian models. First, those models can estimate the entire concentration-time curve, including the AUC of vancomycin levels. Second, as popPK simulations were used to evaluate various sampling scenarios [8], this work also provides simulation and evaluation based on different sampling strategies. Third, similar to some commercial Bayesian-guided dosing decision tools like DoseMe [22], PKRNN-2CM can automatically adjust for variations by receiving information from the EHR stream and delivering real-time concentration predictions. However, this work offers several potential advantages over existing models. First, although DoseMe [22] can integrate real-time data from EHR streams, these models use a limited number of features to estimate PK parameters. In contrast, the PKRNN-2CM model incorporates a broader range of features, which could potentially improve the accuracy of parameter estimation. Second, while the simulation in this study used extensive real information from patients, many popPK simulation studies [8] instead use joint distributions of PK parameters alongside population covariate values. Third, one potential impact of this study is the broader applicability of the PKRNN-2CM model beyond vancomycin. By relying on a 2CM PK model, this work could extend beyond vancomycin and support personalized TDM for other medications. Finally, this work opens possibilities for further research into more complex models, such as multicompartment models with three or more compartments, which may improve the prediction of concentration-time curves for TDM applications.

The study conducted has some limitations that should be acknowledged. First, while AUC of vancomycin levels is a key PK marker for vancomycin in clinical practice, the true AUC or MIC value is not available in our real-world dataset due to sparse observations of vancomycin levels. As a result, we relied on simulation to evaluate model performance across the full time–concentration profile. Because the simulated ground truth is generated from PKRNN-2CM–derived data, there is a potential risk of model confirmation bias. To mitigate this limitation, future studies may consider simulating using alternative models. Additionally, Table S1 in Multimedia Appendix 1 includes results of the simulation generated by a PKRNN-1CM model. When trained on data simulated by the PKRNN-1CM, the PKRNN-1CM model outperforms the PKRNN-2CM, though the performance gap narrows significantly with more simulated measurements compared to 2CM generated simulations. Ideally, as denser real-world datasets become available, they will provide a more robust gold standard for model validation. Second, the study used 48 input variables to obtain patient-specific information compared to most currently available Bayesian models for vancomycin. This may potentially pose the concern of the unavailability of input variables, even though the variables included in this study are commonly measured in hospitals when vancomycin is used for treating bacterial infections. While the inclusion of multiple variables enhances model personalization, it may reduce interpretability. Future work could include feature attribution or other interpretable modeling approaches to clarify how key variables contribute to the estimation of PK parameters and improve clinical insight. Furthermore, we only simulated the uniform infusion rate of 1 g/h used in the current PKRNN-2CM model settings, as this is the default setting used in MHHS. However, the PKRNN-2CM model does not need to assume this fixed parameter and can incorporate other infusion rates as well. Finally, this study primarily focuses on developing the PKRNN-2CM model and comparing it with the PKRNN-1CM model and a baseline Bayesian model. Therefore, a comprehensive comparison with other widely used methods, such as popPK models [22], Bayesian-based methods [5,18], and other deep learning–based models [7,8], is warranted in future studies. This study also focuses solely on the methodological development of vancomycin TDM. To translate this into the clinical practice of model-informed precision dosing, substantial future work on clinical implementation is needed. Although model performance was evaluated in this study, the clinical impact of the PKRNN-2CM model, such as optimizing vancomycin dosing or decreasing side effects based on predicted values, should be evaluated in the future. In practice, the model would be integrated into a model-informed precision dosing system that ingests routinely collected EHR data, generates individualized PK parameter estimates, and provides dose adjustment recommendations for clinicians. Developing and validating such a workflow, including real-time data integration and clinician-facing decision support, is beyond the scope of this study but represents an important direction for future work.

In conclusion, the PKRNN-2CM model was developed, which can be viewed as an improved version of the PKRNN-1CM model. The study demonstrated the superiority of the 2-compartment PK model versus the 1-compartment PK model for vancomycin concentration prediction when using RNN as part of the predictive model. The study also demonstrated that the 2-compartment PK model is significantly better than the 1-compartment PK model when using sparse, irregularly sampled EHR data obtained from the real world. Overall, the findings suggest that the PKRNN-2CM model has the ability to improve the accuracy of vancomycin concentration predictions and could be applied to other PK modeling tasks using time-series EHR data. The results highlight the potential of the PKRNN-2CM model for improving personalized vancomycin TDM in clinical practice.

## Supplementary material

10.2196/81103 Multimedia Appendix 1 Supplementary background and methodological details, variable definitions, and extended simulation results.

10.2196/81103 Multimedia Appendix 2 Mathematical derivation of the pharmacokinetic model.

## Abbreviations

1CM: 1-compartment model
2CM: 2-compartment model
AUC: area under the concentration-time curve
EHR: electronic health record
MHHS: Memorial Hermann Health System
MIC: minimum inhibitory concentration
ODE: ordinary differential equation
PK: pharmacokinetic
PKRNN-1CM: pharmacokinetic recurrent neural network–1 compartment model
PKRNN-2CM: pharmacokinetic recurrent neural network–2 compartment model
popPK: population pharmacokinetic
RMSE: root mean square error
RNN: recurrent neural network
TDM: therapeutic drug monitoring
VTDM: vancomycin therapeutic drug monitoring
