# Supplementary material for: Improving Vancomycin Therapeutic Drug Monitoring With a Deep Learning–Based Two-Compartment Predictive Model: Development and Validation Study
Source: JMIR AI. 2026 Jun 1;5:e81103. doi: 10.2196/81103 (PMC13225945; doi:10.2196/81103)
Supplement: Multimedia Appendix 1 [file ai-v5-e81103-s001.docx]

Supplementary materials

## A Additional background and methodological details

### A1 Background

Therapeutic drug monitoring (TDM) involves measuring a specific drug's blood concentration in a patient's bloodstream at certain intervals to keep it in a safe therapeutic range, allowing individual dosage regimens to be optimized [1]. TDM is primarily used to monitor drugs with narrow therapeutic ranges, drugs with high pharmacokinetic (PK) variability, and drugs with known serious adverse effects. Vancomycin, a widely used antibiotic as primary therapy for infections caused by methicillin-resistant Staphylococcus aureus (MRSA) since 1958, requires TDM to achieve optimal efficacy and avoid toxicity [2].

Traditionally, the PK parameters for vancomycin TDM can be estimated using four major methods, based on [1]: trough monitoring, linear regression, population PK methods, and Bayesian estimation. Trough concentrations, serving as a surrogate marker for the area under the concentration-time curve (AUC) to minimum inhibitory concentration (MIC) ratio, offer a simpler monitoring approach. Despite the ease of use and minimal resource requirements of trough monitoring and population PK methods, PK parameters differ greatly among models, and therefore a single vancomycin model may not be applied to diverse patient populations. While linear regression and Bayesian methods offer more precise dosage regimens to achieve targeted AUC compared to trough-based dosage regimens, they also require additional resources, such as trained healthcare professionals and improved information technology. As explained by [1] , Bayesian methods offer personalized vancomycin dosing recommendations by optimally utilizing both population PK model parameters and patient-specific PK data. In view of this benefit, the most recent national guidelines advocate individualized dosing guided by Bayesian methods for TDM.

It is important to note, however, that Bayesian models still have multiple limitations. The Bayesian model itself is often designed for a specific group of patients and uses the characteristics of their population, therefore not covering other populations effectively [4]. Additionally, these models are typically used in patients with stable PK parameters, and may not be appropriate for patients with unstable clinical conditions, such as patients in intensive care unit (ICU) [3]. Lastly, the Bayesian model often incorporates only a limited number of patient-specific variables while other related factors that could improve the prediction are not taken into account [4].

The determination of the number of compartments in developing population PK models is important for describing the PK of drugs [10]. A PK compartment is a mathematical concept that refers to the space in which a drug is likely to be distributed in the body. Hence, compartment models can be used to simulate vancomycin administration, distribution, and elimination. In a 1CM PK model, the body is assumed to behave as a single, uniform compartment, which implies that vancomycin is distributed evenly throughout the body. In a multi-compartment PK model, vancomycin is initially distributed quickly to the first compartment, followed by a slower elimination phase for redistribution among other compartments. Given that the human body is inherently multi-compartmental, more compartments in the PK model tend to provide a more realistic representation of vancomycin distribution. Therefore, it is expected that more compartments should provide a more accurate description of the change in vancomycin concentration. Vancomycin PK has been described using one-, two-, and three-compartment models, while most Bayesian forecasting programs utilize one- or two-compartment models [10].

While historical practices and some current studies favor the simplicity of 1CM models, recent evidence suggests the superiority of two-compartment (2CM) models in many aspects. A 2CM model is generally considered to describe vancomycin PK [10]. Evidence from studies [10,14,15] reinforces the advantages of 2CM models over 1CM models. For example, the linear regression equations reveal significant differences in predicting maximum serum concentrations, emphasizing a 61% underestimation with the 1CM model [10].

### A2 Simulation framework

The framework can be technically described into 7 steps:

| **Pseudocode: Simulation Framework** |
| --- |
| **Step 1: Load the real-world data**  **Step 2: Train the PKRNN-2CM model and get predicted concentrations by time steps**  PKRNN-2CM_model = train_model(PKRNN-2CM, real_data)  predicted_concentrations = PKRNN-2CM_model. predict_concentrations(real_data)  **Step 3: Evaluate derived model performance using RMSE**  if Simulation == False:  RMSE = calculate_RMSE(observed_concentrations, predicted_concentrations)  **Step 4: Simulation process**  if Simulation == True:  duplicated_data = duplicate_file(real_data)  calculated_concentrations = calculate_concentrations(predicted_concentrations)  simulated_data = modify_data(duplicated_data, calculated_concentrations, simulation_options)  **Step 5: Train the PKRNN-1CM-estimated or PKRNN-2CM-estimated model on simulated data**  estimated_model = train_model(estimated_model_type, simulated_data)  predicted_concentrations_ estimated = estimated_model.predict_concentrations(simulated_data)  **Step 6: Evaluate estimated model performance using RMSE**  RMSE_ estimated = calculate_RMSE(simulated_labels, predicted_concentrations_estimated)  **Step 7: Compare RMSEs of PKRNN-1CM and PKRNN-2CM**  if estimated_model_type == PKRNN-1CM:  PKRNN-1CM_RMSE_estimated = RMSE_estimated  elif estimated_model_type == PKRNN-2CM:  PKRNN-2CM_RMSE_estimated = RMSE_estimated |

RMSE: Root Mean Square Error


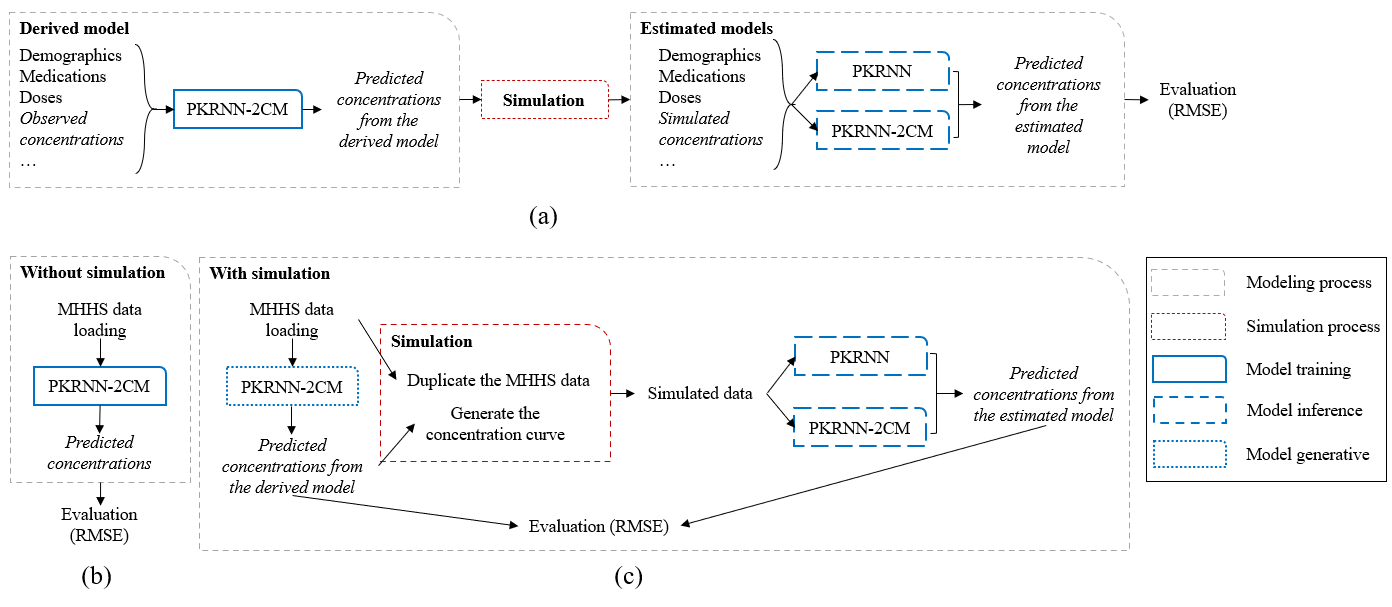


The top figure (a) shows the sequential steps: training the PKRNN-2CM model on MMHS data, generating simulated datasets, running the PKRNN-1CM-estimated or PKRNN-2CM-estimated model, and evaluating model performance. The bottom figures provide (b) detailed descriptions of the PKRNN-2CM training framework without simulation, (c) the simulation process.

**Figure A.1 Simulation framework**

Figure A.1 illustrates the simulation framework employed in this study. Figure A.1(a) demonstrates the sequential steps involved: firstly, training a PKRNN-2CM model on MMHS data to establish the PKRNN-2CM-derived model for simulation. Subsequently, the predicted concentrations are utilized to generate simulated datasets. These simulated concentrations, along with other patient information, are then inputted into the PKRNN-1CM-estimated or PKRNN-2CM-estimated model. The final step involves evaluating the performance of the models by comparing the predicted concentration-time curve derived from the PKRNN-2CM-derived model with that obtained from the PKRNN-1CM-estimated or PKRNN-2CM-estimated model. The bottom two figures provide a detailed depiction of the aforementioned framework. During model execution, there are two possible scenarios represented in the bottom figures. If the simulation parameter is set to false (Figure A.1(b)), the process involves three steps: loading the MMHS data, executing the PKRNN-2CM model, and obtaining the predicted concentration. Conversely, if the simulation parameter is set to true (Figure A.1(c)), the simulation commences after acquiring the predicted concentration from the PKRNN-2CM-derived model. Specifically, the MMHS data file is duplicated, and concentrations are calculated to generate the predicted concentration-time curve. The core simulation process entails utilizing the calculated concentrations to modify the previous labels and other relevant data based on different simulation options. Subsequently, the estimated model are executed using the simulated data, and predicted concentrations are obtained, followed by model evaluation.

### A3 Simulation and evaluation process overview

Figure A.2 provides a comprehensive description of the simulation and evaluation process. The simulation moves measurements from the real-world dataset to target locations (peak or trough) Two sub-options were considered: adding measurements to randomly selected half doses of the patient or adding measurements for every dose. Three location options were explored: peak, trough, and both, where the "both" option means simulating measurements at both peak and trough levels of the concentration-time curve. Evaluation criteria were also diverse, permitting assessment at peak, trough, or both locations.
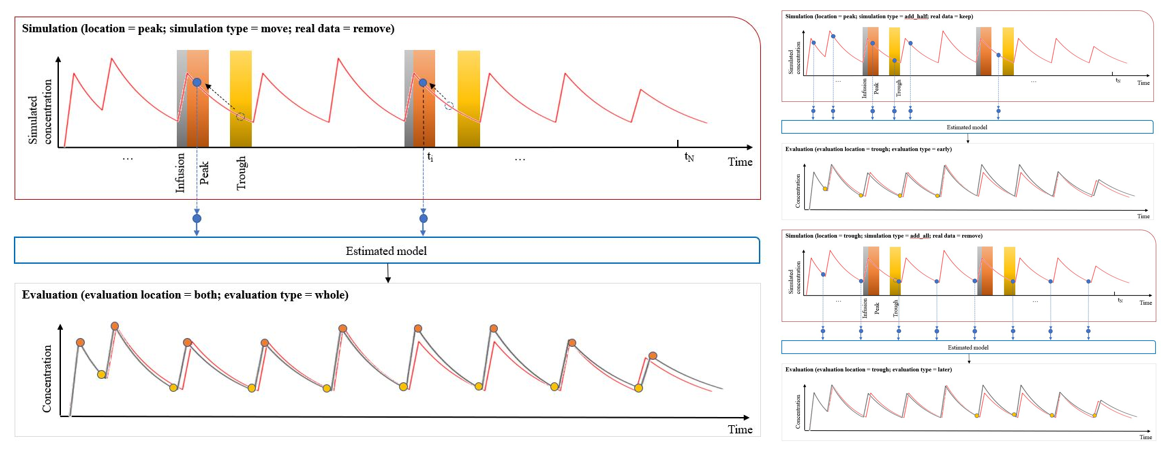


**Figure A.2 Simulation and evaluation options overview**

## B Generic names of medications included in the model

| **Generic names of medications included in the model** |
| --- |
| abacavir, abacavir/dolutegravir/lamiVUDine, acyclovir, albumin human, albuterol, albuterol-ipratropium, allopurinol, amikacin, Amino Acids 10% (Premasol), Amino Acids 3% w/lytes (Procalamine), AMIODarone, amitriptyline, amLODipine, amoxicillin, amoxicillin-clavulanate, amphotericin B liposomal, ampicillin, ampicillin-sulbactam, aspirin, atazanavir, atenolol, atorvastatin, atovaquone, atropine, azathioprine, azithromycin, aztreonam, benazepril, bictegravir/emtricitabine/tenofovir, bosentan, buPROPion, busPIRone, calcitriol, calcium acetate, calcium carbonate, calcium carbonate-magnesium chloride, calcium chloride, calcium gluconate, calfactant, captopril, carbamazepine, carbidopa/entacapone/levodopa, carbidopa-levodopa, CARboplatin, carboprost, carvedilol, cefazolin, cefdinir, cefepime, cefoxitin, cefpodoxime, ceftaroline, ceftazidime, ceftazidime-avibactam, ceftolozane-tazobactam, ceftriaxone, cefuroxime, celecoxib, cephalexin, chlorproMAZINE, cholecalciferol, cholestyramine, cilostazol, ciprofloxacin, clarithromycin, clindamycin, clonidine, clopidogrel, cobicistat-darunavir, colchicine, colistimethate, cycloSPORINE, cycloSPORINE ophthalmic, dabigatran, dapsone, daptomycin, darunavir, desmopressin, dexamethasone, Dextrose 10% in Water IV, Dextrose 20% in Water IV, Dextrose 5% in Lactated Ringers IV, Dextrose 5% in Water IV, Dextrose 5% with 0.225% NaCl IV, Dextrose 5% with 0.45% NaCl IV, Dextrose 5% with 0.9% NaCl IV, Dextrose 50% in Water IV, Dextrose 70% in Water IV, diazepam, diclofenac, digoxin, diltiazem, diphenhydrAMINE, DOBUTamine, dolutegravir, donepezil, DOPamine, doxazosin, DOXOrubicin, doxycycline, efavirenz, emtricitabine, emtricitabine-tenofovir, enalapril, enoxaparin, EPINephrine, epoetin alfa, epoprostenol, ergocalciferol, ertapenem, erythromycin, esmolol, ethambutol, etoposide, etravirine, famciclovir, famotidine, febuxostat, fenofibrate, FENTanyl, fidaxomicin, finasteride, flecainide, fluconazole, fondaparinux, foscarnet, fosfomycin, furosemide, gabapentin, ganciclovir, gentamicin, glimepiride, glipiZIDE, glucagon, hepatitis B immune globulin, hydrALAZINE, hydrochlorothiazide, hydrocortisone, hydroxychloroquine, hydroxyurea, hydrOXYzine, ibuprofen, ibutilide, ifosfamide, imatinib, infliximab, insulin glargine, insulin isophane, insulin isophane-insulin regular, insulin lispro, insulin lispro-insulin lispro protamine, Insulin regular, irbesartan, isoniazid, isosorbide dinitrate, isosorbide mononitrate, itraconazole, ketamine, ketorolac, labetalol, lacosamide, lamivudine, leflunomide, leucovorin, levalbuterol, levetiracetam, levofloxacin, linezolid, lisinopril, lithium, loperamide, loratadine, LORAzepam, losartan, lubiprostone, magnesium citrate, magnesium gluconate, magnesium hydroxide, magnesium lactate, magnesium oxide, magnesium sulfate, mannitol, medroxyPROGESTERone, megestrol, melatonin, meloxicam, meropenem, mesalamine, metFORmin, methadone, methotrexate, methylnaltrexone, methylphenidate, methylPREDNISolone, metoclopramide, metoprolol, METRONIDazole, micafungin, midazolam, midodrine, milrinone, minocycline, minoxidil, mirtazapine, mycophenolate mofetil, mycophenolic acid, nafcillin, naloxone, naproxen, nevirapine, niacin, niCARdipine, NIFEdipine, nitazoxanide, nitrofurantoin, norepinephrine, nortriptyline, octreotide, olanzapine, olmesartan, omega-3 polyunsaturated fatty acids, omeprazole, ondansetron, oseltamivir, oxcarbazepine, oxybutynin, OXYcodone, oxytocin, pamidronate, pantoprazole, paroxetine, pembrolizumab, penicillin G benzathine, penicillin G potassium, penicillin V potassium, pentamidine, phenobarbital, phenytoin, pioglitazone, piperacillin-tazobactam, polymyxin B sulfate, potassium acetate, potassium chloride, potassium phosphate, potassium phosphate-sodium phosphate, pravastatin, prazosin, prednisoLONE, predniSONE, pregabalin, primaquine, probenecid, prochlorperazine, progesterone, promethazine, propofol, propranolol, pyrazinamide, pyridoxine, quetiapine, quinine, raltegravir, ramipril, ranitidine, repaglinide, ribavirin, rifabutin, rifampin, rifaximin, ritonavir, rivaroxaban, rosuvastatin, sertraline, sevelamer, sildenafil, simvastatin, sirolimus, sitagliptin, sodium acetate, sodium bicarbonate, sodium biphosphate-sodium phosphate, sodium chloride, Sodium Chloride 0.45% IV, Sodium Chloride 0.9% IV, Sodium Chloride 3% IV, sodium chloride nasal, sodium citrate, sodium ferric gluconate complex, sodium hypochlorite topical, sodium phosphate, sodium polystyrene sulfonate, sotalol, spironolactone, sulfasalazine, sumatriptan, tacrolimus, tamsulosin, tenofovir, terazosin, terbinafine, testosterone, ticagrelor, tigecycline, tizanidine, tobramycin, tolvaptan, topiramate, torsemide, TPN Central Order Details - Neonatal, TPN Order Details - Pediatric, TPN Peripheral Order Details - Neonatal, tramadol, trazodone, valacyclovir, valganciclovir, valproic acid, valsartan, vancomycin, vasopressin, verapamil, vinCRIStine, voriconazole, warfarin, zidovudine |

## C Continuous variables included in the model

| **Type of Features** | **Features** |
| --- | --- |
| Time | Time (measured in days from the start of the encounter), Time elapsed since last event |
| Demographic data | Age at the time of visit, Gender, Ethnicity/Race, Height, Weight |
| Vital signs | Heart rate, Body temperature, Systolic blood pressure, Diastolic blood pressure, Respiratory rate, SpO2, O2 flow |
| Vancomycin dose | Vancomycin dose |
| Laboratory results | White blood cell count, Hematocrit, Hemoglobin, Red blood cell counts, Platelet counts, Neutrophils #, Lymphocytes (%), Lymphocytes counts, Monocytes (%), Monocytes counts, Eosinophils (%), Eosinophils counts, Basophils (%), Basophils counts, Nucleated red blood cell (%), Sodium level, Potassium level, Creatinine level, Blood urea nitrogen level, Estimated glomerular filtration rate, Bicarbonate level, Chloride level, Glucose level, Total calcium level, Phosphorus level, Magnesium level, Albumin level, Total bilirubin level, Total protein level |
| Vancomycin concentration | Vancomycin concentration |

## D VTDM RMSE on simulated data

| **Simulation Types** | **RMSE** |
| --- | --- |
| Peak Add all | 329.39 |
| Peak Add half | 244.93 |
| Trough Add all | 4.49 |
| Trough Add half | 5.31 |
| Both Add all | 39.65 |
| Both Add half | 26.17 |

| **Add measurements for half doses** | **Average RMSE (STD)** | | | | ***P*** | | |
| --- | --- | --- | --- | --- | --- | --- | --- |
| **Simulation location** | **PKRNN-1CM-estimated** | | | **PKRNN-2CM-estimated** | | |  |
| Peak^*^ | 3.04 (0.43) | 4.91 (0.45) | | | <.001 | | |
| Trough^**^ | 3.08 (0.31) | 11.02 (3.39) | | | <.001 | | |
| Both^***^ | 2.81 (0.18) | 4.38 (0.31) | | | <.001 | | |
| **Add measurements for all doses** | **Average RMSE (STD)** | | | | ***P*** | | |
| **Simulation location** | **PKRNN-1CM-estimated** | | **PKRNN-2CM-estimated** | | |  | |
| Peak^*^ | 2.46 (0.16) | 3.64 (0.39) | | | <.001 | | |
| Trough^**^ | 2.95 (0.18) | 7.09 (1.74) | | | <.001 | | |
| Both^***^ | 2.35 (0.24) | 2.87 (0.11) | | | <.001 | | |

**Table D.1 Simulation results for datasets generated by a PKRNN-1CM model**
