# Supplementary material for: Improving Vancomycin Therapeutic Drug Monitoring With a Deep Learning–Based Two-Compartment Predictive Model: Development and Validation Study
Source: JMIR AI. 2026 Jun 1;5:e81103. doi: 10.2196/81103 (PMC13225945; doi:10.2196/81103)
Supplement: Multimedia Appendix 2 [file ai-v5-e81103-s002.pdf]

Analytical Solution of the Two-Compartment ODE

Regular (Eigen-decomposition) and Sylvester (Matrix Exponential) Forms

1 Problem Statement

The two-compartment pharmacokinetic model is described by:

$$\frac{dC_A}{dt} = \frac{I}{V_1} - \frac{CL}{V_1} C_A - \frac{Q(C_A - C_B)}{V_1}, \tag{1}$$

$$\frac{dC_B}{dt} = \frac{Q(C_A - C_B)}{V_2}, \tag{2}$$

where C_A and C_B are the drug concentrations in the central and peripheral compartments, V_1 and V_2 are the compartment volumes, CL is the (total body) clearance from the central compartment, Q is the inter-compartmental clearance, and I is the infusion rate (mass per unit time, nonzero only during infusion).

2 Matrix Formulation

Define the state vector and input:

$$\mathbf{x} = \begin{pmatrix} C_A \\ C_B \end{pmatrix}, \qquad \mathbf{b} = \begin{pmatrix} I/V_1 \\ 0 \end{pmatrix}. \tag{3}$$

Equations (1)–(2) become

$$\frac{d\mathbf{x}}{dt} = M\,\mathbf{x} + \mathbf{b}, \tag{4}$$

with the system matrix

$$M = \begin{pmatrix} -\dfrac{CL+Q}{V_1} & \dfrac{Q}{V_1} \\ \dfrac{Q}{V_2} & -\dfrac{Q}{V_2} \end{pmatrix} \equiv \begin{pmatrix} m_{11} & m_{12} \\ m_{21} & m_{22} \end{pmatrix}. \tag{5}$$

3 Solution I — Regular (Eigen-decomposition) Form

3.1 Eigenvalues

The eigenvalues of M are

$$\lambda_{1,2} = \frac{\operatorname{tr}(M) \mp \Delta}{2}, \qquad \Delta = \sqrt{\operatorname{tr}(M)^2 - 4\det(M)}, \tag{6}$$

where

$$\mathrm{tr}(M) = m_{11} + m_{22} = -\frac{CL + Q}{V_1} - \frac{Q}{V_2}, \tag{7}$$

$$\det(M) = m_{11}\, m_{22} - m_{12}\, m_{21} = \frac{CL \cdot Q}{V_1 \cdot V_2}. \tag{8}$$

Because $CL, Q, V_1, V_2 > 0$, both eigenvalues are real and negative ($\lambda_1 < \lambda_2 < 0$).

3.2 Modal Decomposition

We decompose C_A and C_B into two modes:

$$\underbrace{\begin{pmatrix} C_A \\ C_B \end{pmatrix}}_{\mathbf{x}} = \underbrace{\begin{pmatrix} C_3 & C_4 \\ 1 & 1 \end{pmatrix}}_{S} \underbrace{\begin{pmatrix} C \\ D \end{pmatrix}}_{\boldsymbol{\xi}}, \tag{9}$$

where (C, D) are the modal amplitudes in each eigenspace, and the eigenvector coefficients satisfy:

$$C_3 = -\frac{\lambda_1 - m_{22}}{m_{21}} = -\frac{\lambda_1 + Q/V_2}{Q/V_2}, \qquad C_4 = -\frac{\lambda_2 - m_{22}}{m_{21}} = -\frac{\lambda_2 + Q/V_2}{Q/V_2}. \tag{10}$$

3.3 General Solution (Piecewise-Constant Input)

During each dosing interval the input I is constant. The modal equations decouple:

$$\dot{C} = \lambda_1\, C + \tilde{b}_1, \qquad \dot{D} = \lambda_2\, D + \tilde{b}_2, \tag{11}$$

where $\tilde{\mathbf{b}} = S^{-1}\mathbf{b}$.

The particular (steady-state) solutions are constants:

$$C_1 = -\frac{\tilde{b}_1}{\lambda_1} = \frac{r\, m_{21}}{\lambda_1\, \Delta}, \qquad C_2 = -\frac{\tilde{b}_2}{\lambda_2} = -\frac{r\, m_{21}}{\lambda_2\, \Delta}, \tag{12}$$

where $r = I/V_1$.

3.4 Infusion + Decay Solution

Infusion phase ($0 \le t \le t_{\mathrm{inf}}$, $I > 0$). Starting from prior modal amplitudes $(C_{\mathrm{prev}}, D_{\mathrm{prev}})$:

$$C(t) = (C_{\mathrm{prev}} + C_1)\, e^{\lambda_1 t} - C_1, \tag{13}$$

$$D(t) = (D_{\mathrm{prev}} + C_2)\, e^{\lambda_2 t} - C_2. \tag{14}$$

At the end of infusion define:

$$C_{\mathrm{peak}} = (C_{\mathrm{prev}} + C_1)\, e^{\lambda_1 t_{\mathrm{inf}}} - C_1, \qquad D_{\mathrm{peak}} = (D_{\mathrm{prev}} + C_2)\, e^{\lambda_2 t_{\mathrm{inf}}} - C_2. \tag{15}$$

Decay phase ($0 \le s \le t_{\mathrm{dec}}$, $I = 0$). With $t_{\mathrm{dec}} = t_d - t_{\mathrm{inf}}$:

$$C(s) = C_{\mathrm{peak}}\, e^{\lambda_1 s}, \tag{16}$$

$$D(s) = D_{\mathrm{peak}}\, e^{\lambda_2 s}. \tag{17}$$

The concentrations at the end of the interval are recovered via (9):

$$\boxed{C_A = C_3\, C(t_{\mathrm{dec}}) + C_4\, D(t_{\mathrm{dec}}), \qquad C_B = C(t_{\mathrm{dec}}) + D(t_{\mathrm{dec}}).} \tag{18}$$

3.5 AUC (Regular Form)

$$\text{AUC}_{\text{inf}} = C_3 \left[\frac{C_{\text{prev}} + C_1}{\lambda_1} \left(e^{\lambda_1 t_{\text{inf}}} - 1 \right) - C_1\, t_{\text{inf}} \right] + C_4 \left[\frac{D_{\text{prev}} + C_2}{\lambda_2} \left(e^{\lambda_2 t_{\text{inf}}} - 1 \right) - C_2\, t_{\text{inf}} \right], \quad (19)$$

$$\text{AUC}_{\text{dec}} = C_3 \frac{C_{\text{peak}}}{\lambda_1} \left(e^{\lambda_1 t_{\text{dec}}} - 1 \right) + C_4 \frac{D_{\text{peak}}}{\lambda_2} \left(e^{\lambda_2 t_{\text{dec}}} - 1 \right). \quad (20)$$

4 Solution II — Sylvester (Matrix Exponential) Form

4.1 Matrix Exponential via Sylvester's Formula

For a 2×2 matrix M with distinct eigenvalues $\lambda_1 \neq \lambda_2$, the matrix exponential is given by the **Sylvester–Lagrange interpolation**:

$$e^{Mt} = \frac{e^{\lambda_1 t}}{\lambda_1 - \lambda_2} (M - \lambda_2 I) - \frac{e^{\lambda_2 t}}{\lambda_1 - \lambda_2} (M - \lambda_1 I). \quad (21)$$

Define $\delta = \lambda_1 - \lambda_2 = -\Delta$ (note $\delta < 0$ since $\lambda_1 < \lambda_2$) and the weight functions

$$w_1(t) = \frac{e^{\lambda_1 t}}{\delta}, \qquad w_2(t) = \frac{e^{\lambda_2 t}}{\delta}. \quad (22)$$

Then the **propagator matrix** $P(t_d) = e^{M t_d}$ has entries:

$$P = \begin{pmatrix} w_1(m_{11} - \lambda_2) - w_2(m_{11} - \lambda_1) & (w_1 - w_2)\, m_{12} \\ (w_1 - w_2)\, m_{21} & w_1(m_{22} - \lambda_2) - w_2(m_{22} - \lambda_1) \end{pmatrix}. \quad (23)$$

4.2 Integral of the Matrix Exponential (Input Response)

The particular solution requires the integral

$$\int_0^{t_{\text{inf}}} e^{M(t_d - s)}\, ds = \int_{t_{\text{rem}}}^{t_d} e^{M\tau}\, d\tau, \qquad t_{\text{rem}} = t_d - t_{\text{inf}}. \quad (24)$$

Applying Sylvester's formula element-wise, the integrated weight functions are:

$$\bar{w}_k = \int_{t_{\text{rem}}}^{t_d} \frac{e^{\lambda_k \tau}}{\delta}\, d\tau = \frac{e^{\lambda_k t_d} - e^{\lambda_k t_{\text{rem}}}}{\delta\, \lambda_k}, \qquad k = 1, 2. \quad (25)$$

When $\lambda_k \to 0$, the limit is $\bar{w}_k \to t_{\text{inf}}\, e^{\lambda_k t_{\text{rem}}}/\delta$.

The input is applied only to the first component: $\mathbf{b} = (I/V_1,\ 0)^\top = (r,\ 0)^\top$ where $r = I/V_1$, and I is taken as 1000 (mg / hr) so $r = 1000/V_1$ per dose.

The first column of the integrated Sylvester matrix gives the input response:

$$\mathbf{g} = \begin{pmatrix} g_1 \\ g_2 \end{pmatrix} = \begin{pmatrix} \bar{w}_1(m_{11} - \lambda_2) - \bar{w}_2(m_{11} - \lambda_1) \\ (\bar{w}_1 - \bar{w}_2)\, m_{21} \end{pmatrix}. \quad (26)$$

4.3 Complete Update Rule

The state at the end of an interval of total duration t_d (containing an infusion of duration t_{inf}) is:

$$\boxed{\mathbf{x}(t_d) = P(t_d)\,\mathbf{x}(0) + \mathbf{g}\,\frac{I}{V_1}} \tag{27}$$

or component-wise:

$$C_A(t_d) = p_{11}\,C_A(0) + p_{12}\,C_B(0) + g_1\,\frac{I}{V_1}, \tag{28}$$

$$C_B(t_d) = p_{21}\,C_A(0) + p_{22}\,C_B(0) + g_2\,\frac{I}{V_1}. \tag{29}$$

4.4 Remark on $Q = 0$

The Sylvester form can handle the degenerate case $Q = 0$ (no inter-compartmental transfer), since the limiting forms of $\bar{w}_k$ in Eq. (25) remain well-defined when an eigenvalue approaches zero. However, the additional branching required to evaluate these limits generally makes it slower than the regular form in practice.

5 Summary of Notation

Symbol	Description
C_A	Drug concentration in central compartment
C_B	Drug concentration in peripheral compartment
V_1	Volume of central compartment
V_2	Volume of peripheral compartment
CL	Total body clearance from central compartment
Q	Inter-compartmental clearance
I	Infusion rate (mass/time)
r	$= I/V_1$, infusion rate normalized by central volume
$\lambda_{1,2}$	Eigenvalues of M
Δ	$= \sqrt{\text{tr}(M)^2 - 4\det(M)}$, discriminant
δ	$= \lambda_1 - \lambda_2$, eigenvalue gap
C_3, C_4	Eigenvector ratios (regular form)
P	Propagator matrix e^{Mt_d} (Sylvester form)
$\mathbf{g}$	Input response vector (Sylvester form)
t_d	Total interval duration
t_{inf}	Infusion duration within the interval
t_{rem}	Remaining (decay) time, $= t_d - t_{\text{inf}}$
